# Soziotechnische Innovationen für Sorgegemeinschaften: Gelingensbedingungen für partizipative und integrative Technikentwicklung

**DOI:** 10.1007/s00391-023-02251-7

**Published:** 2023-10-17

**Authors:** Tobias Wörle, Michael Schaller, Florian Fischer

**Affiliations:** Bayerisches Zentrum Pflege Digital, Hochschule für angewandte Wissenschaften Kempten, Albert-Einstein-Str. 6, 87437 Kempten, Deutschland

**Keywords:** Soziale Partizipation, Co-Kreation, Informelle Pflege, Digitalisierung, Ambulante Versorgung, Social participation, Co-creation, Informal care, Digitalization, Home care

## Abstract

Mit dem steigenden Bedarf an pflegerischer Unterstützung im häuslichen Umfeld geht zugleich eine Abnahme in der Bereitschaft sowie den Möglichkeiten zur familialen Pflege einher (u. a. aufgrund gesellschaftlicher Fragmentierung und Individualisierung). Daher bedarf es neuer Formen sozialer Unterstützungsnetzwerke (Sorgegemeinschaften), in denen professionelle Akteure gemeinsam mit informell Pflegenden und Ehrenamtlichen aktiv sind. Hier kann die Digitalisierung ein Instrument darstellen, das in der Gestaltung und Koordination von Pflegearrangements für ältere Menschen auf sozialräumlicher Ebene zu unterstützen vermag. Grundlegende Voraussetzung ist jedoch, dass entsprechende Technologien zum einen partizipativ und zum anderen integriert in bestehende Strukturen entwickelt werden. Das bedeutet, dass nicht nur die Bedürfnisse und Bedarfe der zukünftigen Nutzer:innen umfassend in den Entwicklungsprozess einbezogen werden, sondern diese auch zu aktiv Mitentscheidenden werden. Darüber hinaus sollte sich die entwickelte Technik an bestehenden Versorgungsstrukturen und sozialräumlichen Gegebenheiten orientieren. Dieser konzeptionelle Beitrag nimmt sich dieser beiden Anforderungen an und zeigt anhand eines konkreten Fallbeispiels aus einem partizipativen Technikentwicklungsprojekt auf, wie soziotechnische Innovationen für und mit Sorgegemeinschaften so entwickelt, implementiert und nutzbar gemacht werden können, dass sie nachhaltig wirksam werden.

## Abstract (en)

The increasing need for nursing support in the home environment is accompanied by a decline in the willingness and possibilities for family care (among other things, due to social fragmentation and individualization). New forms of social support networks (caring communities) are therefore needed in which professional players collaborate together with informal caregivers and volunteer helpers. In this context, digitalization can be an instrument that can support the design and coordination of care arrangements for older people at the sociospatial level; however, a fundamental prerequisite is that the corresponding technologies are developed in a participatory manner and integrated into existing structures. This means not only that the needs and requirements of future users are comprehensively included in the developmental process but also that they become active co-decision makers. Furthermore, the developed technology should be oriented to established care structures and sociospatial conditions. This conceptual contribution addresses these two requirements and uses a concrete case study from a participatory technology development project to show how sociotechnical innovations for and with caring communities can be developed, implemented and made usable in such a way that they become sustainably effective.

Ausgehend von soziohistorisch geprägten gesellschaftlichen Veränderungsprozessen sind mannigfaltige Folgen für die Versorgung älterer Menschen mit Hilfe‑, Pflege- und Unterstützungsbedarf zu beobachten. Mittlerweile werden in vielen Strategiepapieren zur Zukunft der Altenhilfe vermehrt Ideen der Sozialraumorientierung bzw. quartiersnahen Versorgung berücksichtigt, um sowohl objektiven Gegebenheiten als auch subjektiven Bedarfen, Bedürfnissen und Interessen gerecht zu werden.

In sozialraumbezogenen Versorgungsarrangements, die Autonomie stärken und ein langes Verbleiben älterer Menschen in der eigenen Häuslichkeit ermöglichen sollen, besteht eine Vielfalt an Bedarfen, Akteuren und Ressourcen. Zu beobachten ist eine Pluralisierung von Pflegesettings, an denen primäre (z. B. Familie, Freund:innen), sekundäre (z. B. Selbsthilfegruppen und Verbände) und tertiäre Netzwerke (professionelle Hilfssysteme) beteiligt sind. Die Versorgungslandschaft im Kontext der häuslichen Versorgung älterer Menschen stellt sich vielfach als fragmentiert dar. Sie bietet aber zugleich vielfältige Optionen und Potenziale im Hinblick auf lokales Sozialkapital, um Pflegebedürftige und auch pflegende Angehörige zu unterstützen. Zusammengeführt zu Sorgegemeinschaften kann die (pflegerische) Versorgung älterer Menschen dem Postulat geteilter Verantwortung zwischen Dienstleistenden, Bewohner:innen des Quartiers, Angehörigen und dem Gemeinwesen im Sinne eines Wohlfahrts- oder Bürger:innen-Profi-Mixes folgen. Dadurch kann es älteren Menschen mit Unterstützungsbedarfen ermöglicht werden, weiter in der vertrauten Umgebung zu leben. Des Weiteren können Eigeninitiative und gegenseitige Hilfe mobilisiert werden – um den Wechsel von einer „Versorgungsgesellschaft“ zu einer „Mitwirkungsgesellschaft“ zu schaffen.

## Sozialraumorientierung und Zusammenarbeit

Um eine bestmögliche Versorgung pflege- und unterstützungsbedürftiger älterer Menschen zu erreichen, bedarf es der Zusammenarbeit. In diesem Zusammenhang können digitale Lösungen zu Vernetzung, Kommunikation und Koordination unterstützend eingesetzt werden. Eine Neuorientierung in der Ausrichtung sozialer Infrastruktur stellt die Etablierung bzw. insbesondere Zusammenführung entsprechender Versorgungsangebote dar, um auf kleinräumiger Ebene generationenübergreifende Strukturen zu bilden sowie Eigeninitiative, Eigenverantwortung und Solidarität der lokalen Bevölkerung zu stärken [7]. Dazu sind vielfach keine neuen Einzelinfrastrukturmaßnahmen zu planen, sondern die verschiedenen Akteure, beginnend mit pflegenden Angehörigen, bis hin zu Akteuren aus Versorgung, Bildung, Verwaltung, sozialer Arbeit und Kultur unter Bezug auf ein bestimmtes Areal (z. B. einen Stadtteil oder eine Versorgungsregion) und Thema, Problem oder Handlungsfeld (z. B. Pflege und Sorge im Alter) zielgerichtet zu vernetzen [7]. Dies kann unterstützt werden durch digitale Instrumente, die es ermöglichen, bestehende Angebote der Versorgung und Unterstützung im Kontext von Alter(n) und Pflege auf sozialräumlicher Ebene zusammenzuführen.

Um eine bedarfs- und bedürfnisgerechte digital unterstützte Vernetzung zu erreichen, sollten zum einen die an der Versorgung beteiligten Akteure frühzeitig in die (Weiter‑)Entwicklung der digitalen Lösung oder deren Anpassung an die lokalen Besonderheiten eingebunden werden. Zum anderen sollte die Technologie in bestehende Infrastrukturen (sowohl vor Ort als auch ggf. mit Blick auf übergreifende Systeme, wie z. B. die Telematikinfrastruktur oder digitale Gesundheits- oder Pflegeanwendungen [DiGA/DiPA]) integriert werden. Partizipative Technikentwicklung bietet eine Möglichkeit, die Perspektiven der beteiligten Akteure frühzeitig und umfassend einzubeziehen in die Ausgestaltung soziotechnischer Innovationen, die zugleich an bestehenden Versorgungsstrukturen (z. B. im Rahmen integrierter Versorgung) und sozialräumlichen Gegebenheiten ansetzen. Darauf weist dieser Beitrag hin und zeigt anhand eines konkreten Fallbeispiels auf, wie soziotechnische Innovationen für und mit Sorgegemeinschaften so entwickelt, implementiert und nutzbar gemacht werden können, um nachhaltig wirksam zu sein.

## Sorgegemeinschaften als neue Formen sozialer Unterstützungsnetzwerke

Zum Begriff der „Sorgegemeinschaft“ bzw. „Caring Community“ selbst liegt in Forschung und Praxis kein einheitliches Verständnis vor. Gerade dessen Offenheit wird mitunter auch als eine seiner Stärken angesehen [14]. Im Querschnitt der damit verbundenen Diskurse (im Überblick beispielsweise Sempach et al. [10]) erscheint der Begriff eher als heuristisches „Sensitizing Concept“ und als sozialpolitisches Ideal denn als wissenschaftlich geschärftes Konzept. Ausgehend von einem weit gefassten Verständnis von Sorge übernehmen in dieser Vorstellung informelle, semiprofessionelle und professionelle Akteure vor Ort gemeinsam Verantwortung, um An- und Zugehörige mit Pflege- und Sorgeaufgaben zu unterstützen. In dem vorliegenden Beitrag werden Sorgegemeinschaften fokussiert auf die Unterstützung älterer Menschen in der eigenen Häuslichkeit in den Blick genommen. Grundsätzlich ist dieses Konzept aber nicht auf ein spezifisches Anwendungsfeld beschränkt, sondern kann sich ebenso auf Felder der Kinder- und Jugendhilfe, der Unterstützung von Menschen mit Behinderung, der Quartiersentwicklung oder andere Handlungskontexte beziehen [9].

Werden mit Sorgegemeinschaften auch Empowerment und Mitbestimmungschancen verbunden, bestehen zugleich kritische Perspektiven. So wird davor gewarnt, Sorgegemeinschaften als zivilgesellschaftlichen „Lückenfüller“ und Substitut öffentlicher Daseinsvorsorge zu funktionalisieren [6, 9, 11]. Konsens besteht allerdings dahingehend, dass im Kontext von Pflege und Sorge in Form von Sorgegemeinschaften verschiedene Akteure gemeinsam Verantwortung innerhalb eines bestimmten Orts, einer Nachbarschaft, eines Quartiers oder einer ganzen Versorgungsregion übernehmen. Von einem weit gefassten Sorgeverständnis ausgehend, bündeln sie, darauf bezogen, nicht nur ihre Aktivitäten und Ressourcen, sondern teilen in unterschiedlichem Umfang auch gemeinsame Wertarchitekturen und Handlungskulturen [9, 14].

## Soziotechnische Innovationen in Sorgegemeinschaften co-kreativ entwickeln

Im Kontext häuslicher Pflege sollen Sorgegemeinschaften mit ihren Unterstützungsressourcen zu Gesundheit und Lebensqualität von Gepflegten und ihren Angehörigen beitragen. Doch sie benötigen ihrerseits Unterstützung, um Entlastungsangebote aufeinander abzustimmen und kontinuierlich weiterzuentwickeln. Hier können unterstützende Informations- und Kommunikationstechnologien ansetzen. In partizipativen oder co-kreativen Technikentwicklungsprojekten fungieren pflegende Angehörige teilweise als „Fürsprecher:innen“ der Pflegebedürftigen, die aufgrund ihres Unterstützungsbedarfs häufig selbst nicht in entsprechende Forschungs- und Entwicklungsvorhaben eingebunden werden (können). In dem im Folgenden beschriebenen Fallbeispiel werden informell Pflegende jedoch als Teil einer Sorgegemeinschaft als zu beteiligende Zielgruppe adressiert.

Im Projekt KoordinAID^1^ verfolgte vor diesem Hintergrund ein interdisziplinäres wissenschaftliches Team gemeinsam mit pflegenden Angehörigen und verschiedenen Akteuren einer Sorgegemeinschaft das Ziel, einen integrierten Versorgungsansatz für die ländlich geprägte Region des Kinzigtals zu entwickeln. Durch technische Unterstützung – mit organisatorischer Anbindung an die Strukturen der Gesundes Kinzigtal GmbH (als regionaler und populationsorientierter Partnerin für die Gesundheitsversorgung) – sollten zwei Anforderungen gelingen: Entlastende Ressourcen in privaten Netzwerken (z. B. durch die Einbindung weiterer Familienmitglieder oder von Nachbar:innen) sollten stärker sichtbar gemacht werden, und die Sorgegemeinschaft sollte befähigt werden, Unterstützungs- und Beratungsangebote besser zu koordinieren und bedarfsgerecht weiterzuentwickeln. Daraus sollte ein soziotechnisches Gesamtkonzept für die Entwicklung, Testung und Implementierung eines neuen, softwareunterstützten Beratungsmodells für die Modellregion hervorgehen. Die im Folgenden beschriebenen Methoden sowie sich daraus ergebenden Erfahrungen und Erkenntnisse beziehen sich auf die Phase der Erprobungs- und Durchführbarkeitsstudie zur Vorbereitung von konkreten Forschungs- und Entwicklungsarbeiten.

In dieser partizipativ und ergebnisoffen gestalteten Phase wurde für diese Region ein soziotechnisches Gesamtkonzept entwickelt. Es umfasst den Aufbau und die Erprobung eines koordinierten, trägerunabhängigen Beratungsansatzes, in dem u. a. eine neue Software zur Visualisierung häuslicher Pflegenetzwerke nicht nur bei der Beratung von Angehörigen, sondern auch bei der Weiterentwicklung der Angebotslandschaft unterstützt. Neben technischen Anforderungskriterien umfasst das Konzept, das im Weiteren als Grundlage für einen partizipativen Forschungs- und Entwicklungsprozess dienen soll, die gezielte strukturelle Einbettung und Erprobung dieses Visualisierungstools in Beratungsangebote vor Ort. Es beinhaltet zudem eine Weiterentwicklung der nötigen Organisationsstrukturen und technischen Infrastrukturen in der Sorgegemeinschaft (u. a. in Form einer beim Landkreis bzw. Pflegestützpunkt angesiedelten Koordinierungsstelle und Anbindung einer Angebotsdatenbank).

An der Erprobungsphase waren neben 4 technischen und 3 wissenschaftlichen Partner:innen informell Pflegende und Sorgegemeinschaftsakteure beteiligt. Die wissenschaftlichen Grundlagen bildeten 7 Fallstudien, in denen über Interviews alltägliche Belastungen, Entlastungsbedarfe und Ressourcennetzwerke pflegender Angehöriger aus der Modellregion auf ihrer „Pflegenden-Reise“ analysiert wurden. Mithilfe von Struktur- und Stakeholder-Analysen wurden u. a. Akteure, Kooperationsbeziehungen, Koordinierungsbedarfe und Unterstützungspotenziale technischer Infrastrukturen in der Sorgegemeinschaft untersucht.

Der Prozess nutzte einen Mix an Methoden und Formaten. Kernelement waren 3 co-kreative Workshops mit pflegenden Angehörigen, Sorgegemeinschaftsakteuren und Partner:innen aus Technik bzw. Wirtschaft und Wissenschaft. In kollaborativen Formaten wie Fokusgruppen, World-Cafés, Design-Thinking-Methoden und Arbeit an Fallszenarien („Pflegenden-Reisen“, Personas, Use Cases) wurden für das angestrebte Versorgungsmodell mögliche Zielszenarien, Lösungswege, Technologieoptionen und -anforderungen in iterativen Schritten gemeinsam erarbeitet. Auch überregionale Expertisen pflegender Angehöriger flossen ein: zum einen durch Online-Konsultationen mit 3 Betroffenenvertretungen, zum anderen wurden pflegende Angehörige als Mitglieder eines vom Fördergeber bestellten Bürger:innenbeirats in die Workshops und über fokusgruppenähnliche Online-Konsultationen eingebunden. Den wissenschaftlichen Partner:innen kam neben fachlichen Aufgaben auch eine moderierende, übersetzende Rolle in diesem Prozess zu.

Im Ergebnis wurde gleichwohl deutlich, dass pflegende Angehörige beim Aufbau und der Stabilisierung ihrer Sorgearrangements Unterstützung und Begleitung wünschen und benötigen – sowohl aus ihrer eigenen Perspektive als auch aus Sicht zentraler Stakeholder der Sorgegemeinschaft. Einerseits besteht der Wunsch, Angebotslücken digital gestützt erkennen und aufgreifen zu können sowie die Angebotslandschaft von den Bedarfen ausgehend gemeinsam weiterzuentwickeln. Andererseits fehlt es in der Sorgegemeinschaft an Werkzeugen, Prozessroutinen und Organisationsstrukturen, die sich dafür eignen und die zugleich den Zusammenhalt und das Zusammenwirken in der Sorgegemeinschaft weiter zu fördern vermögen.

## Erfolgsfaktoren und Gelingensbedingungen

Aus den Erfahrungen bei der Vorbereitung und Umsetzung dieses Prozesses lassen sich einige Bedingungen und Kriterien ableiten; diese erscheinen für die partizipative Entwicklung, Implementierung und auch den Transfer soziotechnischer Innovationen in und mit Sorgegemeinschaften erfolgskritisch. Sie sollen im weiteren Projektdesign handlungsleitend sein und im Rahmen einer projektbegleitend angelegten Evaluation weiter konkretisiert und überprüft werden. Die vermuteten Gelingensbedingungen beeinflussen sich vielfach wechselseitig. Sie sind in diesem Sinne nicht zwingend gegeneinander zu priorisieren. In ihrem Gesamtzusammenhang betrachtet, bilden sie vielmehr wichtige Voraussetzungen dafür, dass soziotechnische Innovationen akzeptiert, nutzbar und letztlich auch wirksam werden können.

Vor dem Hintergrund der Vielfalt an Bedarfen und Bedürfnissen, Akteuren und Ressourcen vermag der Einsatz digitaler Technologien in Sorgegemeinschaften grundsätzlich bis dahin eher fragmentierte Strukturen (z. B. Teilnetzwerke) miteinander zu verbinden und die Bildung produktiver Schnittmengen zwischen heterogenen informellen, semiprofessionellen und professionellen Akteuren zu fördern. Dies kann insbesondere dann gelingen, wenn Koordinierungsvorteile durch die Mediatisierung bzw. digitale Transformation erzielt und genutzt werden können, da soziotechnische Innovationen in Transaktion mit dem Individuum und der Gesellschaft stehen (z. B. indem analoge Interaktionsformen auch in den digitalen Raum ausgelagert oder ausgeweitet werden; [5]). Entsprechende Technologien müssen aber „strukturkonform“, d. h. passfähig zu bestehenden strukturellen Rahmenbedingungen innerhalb der Sorgegemeinschaften und ihrer sozialräumlichen Kontexte, sein – und nicht losgelöst von der realen bzw. „analogen“ (Lebens‑)Umwelt vor Ort. Technische Lösungen müssen beispielsweise kompatibel zu den jeweiligen Organisationsstrukturen, Interaktionsmustern und Abstimmungsprozessen zwischen institutionellen oder anderen Akteuren sein. Es kann zu Problemen oder Widerständen kommen, wenn neue Prozesse der Aushandlung, die sehr zeitaufwendig sind, in den laufenden Arbeitsalltag integriert werden müssen. Insofern sollten technische Lösungen strukturelle bzw. institutionelle Schnittstellen zwischen verschiedenen Netzwerken und Ebenen (z. B. Familien, Quartiersnetzwerke) bilden und Teil einer „systemischen“ Lösung sein, die – im Idealfall – zu einem soziotechnischen Gesamtmodell integriert werden kann. Technik sollte auch keine Konkurrenzen und Dopplungen zu bereits bestehenden Lösungen schaffen, sondern in vorhandene Strukturen integrierbar sein – sowohl auf technischer Ebene der Interoperabilität von IT-Infrastrukturen als auch auf sozialer Ebene zu bereits existierenden Netzwerken oder Angeboten von Einrichtungen und Einzelpersonen.

Davon bleibt unbenommen, dass mit oder durch die Einführung und Nutzung unterstützender digitaler Technologien auch Veränderungen in Strukturen, Organisationen oder Praktiken einhergehen können, mitunter auch sollen. Welche wünschenswerten Potenziale sich über digitale Technologien und soziotechnische Innovationen in der Praxis ergeben könnten, und was hingegen den Beteiligten selbst tatsächlich als attraktiv, machbar oder zumindest akzeptabel erscheint, dazwischen liegt letztlich der mitunter schmale Grat zwischen Akzeptanz und Aneignung oder Ablehnung und Aufgabe technologischer Neuerungen und der mit ihnen potenziell einhergehenden Veränderungen gewohnter Routinen. Da sich Bedürfnisse und Bedarfe auch im zeitlichen Verlauf verändern können, wären iterative Feedback-Loops unter Einbezug aller Stakeholder sinnvoll.

Nicht nur als Teil der Ausgangslage, sondern auch im laufenden Prozess sind angesichts der Heterogenität der Akteure divergierende Interessen zu berücksichtigen. Beispielsweise haben die Nutzer:innen der zu entwickelnden Technik eher den Usability-Aspekt im Fokus, wohingegen die Entwickler:innen evtl. einen anderen Schwerpunkt (z. B. hinsichtlich einer technischen Innovation) setzen. Zudem stellen der im informellen Spektrum mitunter geringe Grad an Institutionalisierung (z. B. zivilgesellschaftlich engagierter Einzelpersonen) und die damit einhergehende Volatilität informeller oder ehrenamtlich getragener Sorgestrukturen für gemeinsame Aktivitäten in Sorgegemeinschaften sowie die Planbarkeit und Kontinuität partizipativer Prozesse gleichermaßen eine Herausforderung dar. Zumeist liegt zunächst ein unscharfes Spektrum an Akteuren vor, das aus einer – anfangs u. U. noch sehr fragilen – Koalition von Willigen, Kümmernden oder treibenden Einzelakteuren besteht [2]. Dabei bleiben – ganz im Sinne der empirischen Offenheit des Sorgegemeinschaftskonzepts – vielfach Fragen offen, wer in welchem Ausmaß und mit welcher Verpflichtung bzw. Verantwortung zu einer Sorgegemeinschaft gehört und wer noch zu beteiligen ist. Hierbei handelt es sich häufig auch um informell Pflegende, die mitunter als eine gesellschaftlich marginalisierte Gruppe klassifiziert werden, deren Perspektive nur selten einbezogen wird, und die selbst kaum (Handlungs‑)Macht haben, ihre Interessen einzubringen und umzusetzen [1]. Auch solche strukturellen Faktoren stellen Herausforderungen für die Steuerbarkeit und Verlässlichkeit von Sorgegemeinschaften dar – diese wiederum können herausfordernd auf die Entwicklung, Implementierung und langfristige Etablierung soziotechnischer Innovationen wirken.

Jenseits solcher strukturbezogenen Faktoren besteht auch das Erfordernis einer „kulturkonformen“ Ausgestaltung soziotechnischer Arrangements, die kompatibel mit den lokalen, soziokulturellen Strukturen, Praktiken und Wissensordnungen sind. Dazu gehört die Berücksichtigung von Zielen, Werten und Handlungskulturen der beteiligten Akteure. Soziokulturelle Tiefendimensionen wie soziohistorisch geprägte lokale Identitäten oder die lokal vorherrschende Kultur der Kooperation und Partizipation können daher von großer Bedeutung sein, ganz davon abgesehen, dass soziotechnische Innovationen und digitale Technologien auch im Einklang mit allgemeinen soziokulturellen und ethischen Standards stehen müssen. So sollten z. B. zur Vermeidung der Entstehung neuer Ungleichheiten die Möglichkeiten des Zugangs zu den Innovationen auch gerecht verteilt sein.

Im Hinblick auf nachhaltige Verankerung und Transfer können neben den strukturellen auch die soziokulturellen lokalen Spezifika sowohl Vor- als auch Nachteil sein. Sofern partizipativ mit Sorgegemeinschaften entwickelte soziotechnische Innovationen sehr spezifisch an lokalspezifische Kontextbedingungen angepasst sein müssen, erscheinen sie u. U. nur bedingt in andere lokale oder regionale Kontexte übertragbar. Statt Blaupausen braucht es hier Adaption, die aber wiederum partizipativ, und damit Akzeptanz und Nachhaltigkeit fördernd, gestaltet sein kann.

Partizipative und co-kreative Entwicklung kann insofern einen dreifachen Mehrwert bieten: Zum ersten kann eine Bedarfs- und Bedürfnisorientierung in der Ausgestaltung der soziotechnischen Innovation gewährleistet werden. Dabei kann responsiv auf die Anliegen der beteiligten Akteure eingegangen werden. Zugleich macht eine adaptive Entwicklung es nicht nur notwendig, sondern auch möglich, der Dynamik sich verändernder Bedürfnisse und Bedarfe besser gerecht zu werden. Zum zweiten wirkt Partizipation inklusiv und befähigend, wenn der Zugang zum partizipativen Technikentwicklungsvorhaben transparent, niedrigschwellig und chancengerecht ist. Dadurch kann bereits über den Prozess der Technikentwicklung Empowerment gefördert werden [12]. Transparenz beinhaltet in diesem konkreten Fall die klare Kommunikation und ggf. gemeinsame – und bestenfalls gleichberechtigte – Aushandlung von Aufgaben, Rollen, Grenzen, Einflussmöglichkeiten und der Ziele. Letzteres auch im Sinne eines Konflikten vorbeugenden Erwartungsmanagements bezüglich der Frage, was überhaupt das gemeinsame Ziel bzw. die Lösung sein kann – und was eben nicht. Und zum dritten kann die Betrachtung des sozialen Felds, das die partizipativ angelegten Projekte umgibt (z. B. wie oben angeführt durch die Analyse des Sozialraums und des darin agierenden Netzwerks und seiner Stakeholder) dazu befähigen, die relevanten Akteure und ihre Einflussmöglichkeiten sowie die strukturellen, historisch gewachsenen Kontextbedingungen zu erfassen – seien sie nun ökonomischer, politischer oder soziokultureller Natur [1].

Trotz dieser positiven Konnotationen partizipativer Technikentwicklung sind Fragen von Macht, potenziellen Konflikten und Legitimationen kontinuierlich zu reflektieren. Mit der Heterogenität der Akteure sowie der mit ihnen assoziierten Interessen und Handlungsmöglichkeiten können divergierende Standards, Zielvorstellungen und Logiken einhergehen [4]. Insbesondere Organisationen oder öffentliche Institutionen (z. B. kommunale Akteure) können beispielsweise auch mit einschränkenden Handlungsoptionen oder Pfadabhängigkeiten, die ihnen rechtliche Rahmensetzungen oder ihre institutionellen Logiken auferlegen, konfrontiert sein. Die Teilung von Handlungs- und Entscheidungsmacht sowie individuelle und organisationale Spezifika der beteiligten Akteure beeinflussen auch die konkrete Partizipation und Kooperation. Dementsprechend ist eine erfolgreiche Moderation von Interessen- und Zielkonflikten unabdingbar, wie dies auch anderweitige Erfahrungen im Konfliktmanagement in (kommunalen) Netzwerken und Beteiligungsprozessen deutlich machen [13]. Im dargestellten Projektbeispiel zeigte sich dies beispielsweise im Zuge von teils sehr unterschiedlichen Zielvorstellungen und Anforderungen aus der überregionalen Perspektive des mit bundesweit dafür bestellten Bürger:innen besetzten Beirats (der vonseiten des Fördergebers festgelegt wurde) einerseits und den Bedarfen und Interessen pflegender Angehöriger aus der Zielregion (die von den Projektbeteiligten selbst eingebunden wurden) andererseits. Daraus ergibt sich die Frage, inwieweit die prozessbeteiligten Akteure jeweils überhaupt eine quasineutrale moderierende Rolle einnehmen können bzw. sollen. Im geschilderten Prozessbeispiel fungierten vielmehr die wissenschaftlichen Partner:innen als Initiierende des Prozesses im Sinne intermediärer Akteure und nahmen auch eine Katalysatorenfunktion im gesamten Entwicklungsprozess ein. Auf diesem Wege können akademische Partner:innen ihrem Auftrag im Rahmen der Third Mission gerecht werden, Impulse aus der außeruniversitären Welt aufzunehmen und zugleich Wissen für die Gesellschaft verfügbar zu machen. Es wird deutlich, dass letztlich auch grundlegende Fragen und Herausforderungen in der Verantwortlichkeit, Prozesshoheit sowie Legitimation und Akzeptanz des Gesamtprozesses und seiner Ergebnisse (nicht zuletzt im Hinblick auf die Implementierung) berührt sind.

Um Prozesse der partizipativen und co-kreativen Technikentwicklung und daraus entstehende soziotechnische Innovation Erfolg bringend zu gestalten, bedarf es mit Rückblick auf die genannten Gelingensbedingungen einer Institutionalisierung und strukturellen Verankerung. Diese bildet Verlässlichkeit und wirkt stabilitätsfördernd [8] – zugleich für die Sorgegemeinschaften und auch die soziotechnische Innovation selbst. Mit einer solchen Verankerung hängt auch die Notwendigkeit einer (institutionalisierten) Kernstruktur einschließlich des Personals und weiterer Ressourcen, wie sie häufig nur von institutionellen Akteuren (z. B. einer Kommune) geleistet werden kann, zusammen. Alternativ zu Strukturen in kommunaler Trägerschaft sind freilich auch andere nachhaltige Finanzierungsmodelle wie Genossenschaftsmodelle oder digitale Allmende [3] denkbar. Fragen nach Finanzierungsmodellen und Betriebskonzepten sind frühzeitig zu berücksichtigen, da eine unzureichende Klärung einen häufigen Grund für den Abbruch nach Projektende darstellt. Zu guter Letzt – oder vielleicht sogar als zentrale Voraussetzung – sollten der Nutzen und praktische Mehrwert nicht nur der Lösung, sondern im Idealfall auch des partizipativen Technikentwicklungsprozesses selbst möglichst frühzeitig, klar und für alle erkennbar sein und auch erreichbar wirken.

## Fazit für die Praxis

- Digitale Technologien sollten als soziotechnische Innovationen Teil einer „systemischen“ Lösung sein.
- Partizipative Technikentwicklung für und mit Sorgegemeinschaften ermöglicht eine Bedarfs- und Bedürfnisorientierung in der Ausgestaltung der soziotechnischen Innovation und befähigt zugleich die Sorgegemeinschaften.
- Vertreter:innen aus der Wissenschaft erscheinen prädestiniert für eine quasineutrale Katalysatorenfunktion zur Förderung soziotechnischer Innovationen in Sorgegemeinschaften.
- Bei der Implementierung von Sorgegemeinschaften als heterogene Akteursnetzwerke erscheint die systematische Einbindung professioneller Netzwerkkoordination und (Konflikt‑)Moderation empfehlenswert.

## Footnotes

1 Das Projekt KoordinAID – IT-unterstützte Koordination lokaler Ressourcennetzwerke zur Entlastung pflegender Angehöriger im ländlichen Raum (Laufzeit: 01.08.2022–31.07.2023) wurde vom Bundesministerium für Bildung und Forschung gefördert.
